# Enhanced Terahertz Phase Retrieval Imaging by Unequal Spaced Measurement

**DOI:** 10.3390/s22103816

**Published:** 2022-05-18

**Authors:** Chungui Xing, Feng Qi, Shuxu Guo

**Affiliations:** 1 State Key Laboratory on Integrated Optoelectronics, College of Electronic Science and Engineering, Jilin University, Changchun 130012, China; xingcg16@mails.jlu.edu.cn; 2 Key Laboratory of Opto-Electronic Information Processing, Shenyang Institute of Automation, Chinese Academy of Sciences, Shenyang 110169, China; qifeng@sia.cn; 3 Institutes for Robotics and Intelligent Manufacturing, Chinese Academy of Sciences, Shenyang 110169, China; 4 Key Laboratory of Liaoning Province in Terahertz Imaging and Sensing, Shenyang 110169, China

**Keywords:** terahertz phase imaging, phase retrieval, terahertz measurement, noise suppression

## Abstract

Terahertz lensless phase retrieval imaging is a promising technique for non-destructive inspection applications. In the conventional multiple-plane phase retrieval method, the convergence speed due to wave propagations and measures with equal interval distance is slow and leads to stagnation. To address this drawback, we propose a nonlinear unequal spaced measurement scheme in which the interval space between adjacent measurement planes is gradually increasing, it can significantly increase the diversity of the intensity with a smaller number of required images. Both the simulation and experimental results demonstrate that our method enables quantitative phase and amplitude imaging with a faster speed and better image quality, while also being computationally efficient and robust to noise.

## 1. Introduction

The terahertz waves cover frequencies from 0.1 to 10 THz and act as a bridge between the infrared and microwave spectra. Being one of the last mastered frequency ranges in the electromagnetic spectrum, the nondestructive and nonionizing properties have enabled various potential applications and triggered many research interests in the fields of biomedicine [1,2,3], security and quality control, etc. [4,5,6]. Terahertz imaging creates new opportunities that have been demonstrated in other spectrum regions, such as the terahertz near-field imaging [7], terahertz tomography [8,9], terahertz focal scanning imaging [10], terahertz coded-aperture imaging [11], etc. However, most of these methods are based on the THz radiation generated by the femtosecond pulses, which is complicated and lacks high intensity. On the other hand, the continuous wave (CW) terahertz can be realized by modern electronic devices which are simple, compact and cost effective. Unfortunately, most of those continuous THz imaging systems only measure the intensity information but not phase information which contains higher contrast for transparent objects [12]. Therefore, phase imaging is important and especially useful for THz biomedical inspection.

Terahertz phase imaging was developed from conventional digital holography. For the first time, the off-axis terahertz holography was implemented in a transmission mode by Mahon et al. [13] using a 100 GHz Gunn diode oscillator in 2004. A growing number of research studies were carried out in the following years due to more efficient terahertz sources and detectors being used in this field [14,15]. Compared to off-axis holography, phase retrieval imaging can be utilized with lower power THz source and simple, compact equipment. Moreover, due to the sing-beam and lensless mode, the diffraction effect and power degradation with optical elements are greatly reduced and the image quality is improved. Most of the phase retrieval techniques are originated from the Gerchberg–Saxton (GS) algorithm to recover the complete complex wavefront [16,17] via a numerical iterative algorithm. Nevertheless, the GS iterative algorithm needs prior object information as the iteration constrain and suffers from slow convergence speed. Contrary to the single or double measurement schemes, the multiple-plane phase retrieval (MPPR) algorithm was investigated by virtue of compact implementation [18]. Without the need of the support constrain and the prior knowledge of the project, the method can obtain an optimal convergence and high-accuracy reconstruction by using different scanning strategies [19]. The scheme has been investigated in the THz range [20,21], in which, the multiple planes were recorded with ordered propagation and equal interval distance.

MPPR uses a backward–forward wave propagation scheme which iteratively updates the multiple diffractive patterns that are successively recorded at diverse distances with the same interval step. The key to the success of the phase recovery is to ensure that the measured patterns contain sufficient redundancy and diversity, which requires more measured planes to provide the redundancy information. However, the increasing number of register planes will lead to noise accumulations, resulting in poor reconstruction image quality. Most of the existing terahertz imaging system works in raster scan mode and it takes a long time to capture each image, for example, capturing a set of diffractive patterns takes more than 36 h [20]. Those factors constrain the application of this phase recovery technique in the terahertz imaging field.

In this paper, we present a lensless terahertz phase retrieval technique based on an unequal interval measured scheme (UE-MPPR). Compared with the conventional MPPR method, the main difference is that the measurement plane in the proposed technique is recorded with unequal interval distance. The unequal spaced measurement scheme in phase retrieval has been employed in the quantitative phase imaging method based on the transport of intensity equation (TIE) in microscopy [22,23,24] to reduce the recovery error; however, the unequal spaced measurement used in the MPPR is to obtain the significant intensity variations in adjacent propagation planes, so the unequal spaced measurement scheme from the TIE is not suitable for the MPPR. From investigations in previous works [19], the small span of the wave propagation helps to recover the high frequency of the object and the large span of the wave propagation helps to reconstruct the low-frequency information of the object. To achieve better phase retrieval imaging quality, more measure planes are required in the conventional MPPR technique, which may result in a low convergence speed and the accumulation of noise. Our proposed technique can significantly overcome such disadvantages by setting unequal measured intervals. With the same number of planes and iterations, both simulations and experimental verifications demonstrate that our method can significantly improve reconstruction quality and convergence speed. Additionally, the accumulated noise can be fairly reduced by the unequal interval method.

The structure of the paper is arranged as follows: Section 2 describes the principle of the unequal interval technique, Section 3 and Section 4 demonstrate the details of the simulations and the experiment result, respectively. Finally, some conclusions are given in Section 5.

## 2. Methods

**Figure 1:**
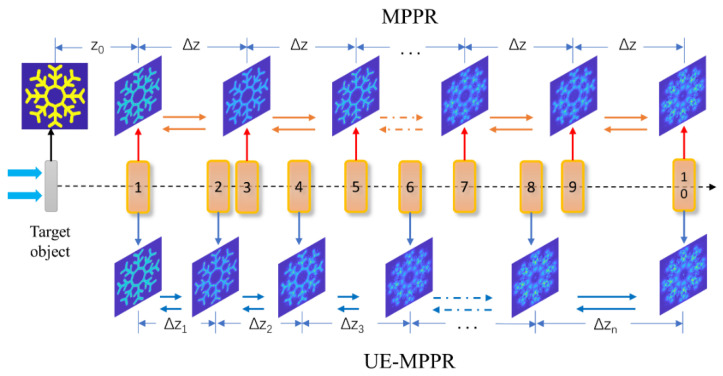
The schematic diagram for the multiple-plane phase retrieval method. Yellow arrows depict the flow of the conventional reconstruction algorithm, blue arrows depict the flow of the proposed algorithm with unequal spaced measurement.

Figure 1 shows the schematic diagram for the recording and reconstruction stages of the terahertz multiple-plane phase retrieval imaging. The sample is placed at the object plane and illuminated by a point source of terahertz radiation, the first measurement plane is located at a distance from the object of $z_{0}$, and the diffracted field distribution set $I_{z}\left(x,y\right)$ was recorded successively by acquiring a sequence of images at multiple distances along the *z* axis through a raster scanning method, the interval space between the rest of the recorded plane is Δz.

The iteration process of the methods begins with a complex wavefield that is constructed from the square root of the first intensity measurement with an initially constant phase field, the wavefront passes through the sequentially measured plane, where the calculated amplitude is replaced by the square root of the measured intensity while retaining the calculated phase. When the wave propagation reaches the last observation plane, it is inversely propagated to the first plane, after which, one iteration is completed. The corresponding calculated complex amplitude is prepared for the next same iteration. Wave propagation is implemented by using the angular spectrum method to solve the Rayleigh–Sommerfeld equation and is given by

(1) $$U_{out}\left(x,y\right)={ℱ}^{-1}\left\{ℱ\left\{U_{in}\left(x,y\right)\right\}e^{jkz\sqrt{1-{\left(\lambda f_{x}\right)}^{2}-{\left(\lambda f_{y}\right)}^{2}}}\right\}$$

where *λ* is the incident terahertz wavelength, k=2π/λ is the wavenumber, z is the distance between the source and the observation plane, $U_{in}$ is the complex field of the input plane, $U_{out}$ is the complex field of the output plane and (x, y) and $\left(f_{x},f_{y}\right)$ are the spatial and frequency coordinates, respectively. ℱ and ${ℱ}^{-1}$ are the direct and inverse Fourier transform. The mean square error (MSE) between the calculated amplitude and measured amplitude is computed per iteration to determine the termination of the iteration. The target complex wavefield can be retrieved by the inverse propagation from the first observation plane to the object plane when all the forward and backward iterations were completed.

(2) $$MSE=\frac{1}{MN}\sum_{x=1}^{M}\sum_{y=1}^{N}{\left[\sqrt{I_{i}\left(x,y\right)}-\left|U_{i}\left(x,y\right)\right|\right]}^{2}$$

Here, i is the iteration number and M and N are the numbers of pixels.

In the conventional MPPR algorithm, sequential propagations, shown in Figure 1 and indicated by yellow arrows, are carried out between the measurement planes with the same interval step Δz. Based on the previous discussion, the process of the iterative complex wavefield reconstruction using intensity distributions is most effective when the diffraction pattern shifts away from one observation plane to another and fully “regenerates”, that means the conventional process with the same propagation interval step cannot fully present the diversity of the wavefield distribution. In general, this interval step corresponds to the size of the speckle. Here, the Fresnel number is introduced into our proposed algorithm to define the interval step [25]. The Fresnel number is used to evaluate the spatial frequency distribution of the diffraction patterns, which is given by:

(3) $$F^{\#}=\frac{D^{2}}{\lambda z}$$

where the D denotes the longitudinal length of the diffraction pattern and the λ is the wavelength of the incident terahertz wave. If the difference in the $F^{\#}$ between the adjacent measurement planes is fixed, then the nonlinear increase in the Δz between the adjacent diffraction patterns follows the variation in the $\Delta F^{\#}$:

(4) $$\Delta F^{\#}=\frac{D^{2}}{\Delta z_{i+1}\times \lambda }-\frac{D^{2}}{\Delta z_{i}\times \lambda }$$

## 3. Numerical Simulation Results

**Figure 2:**
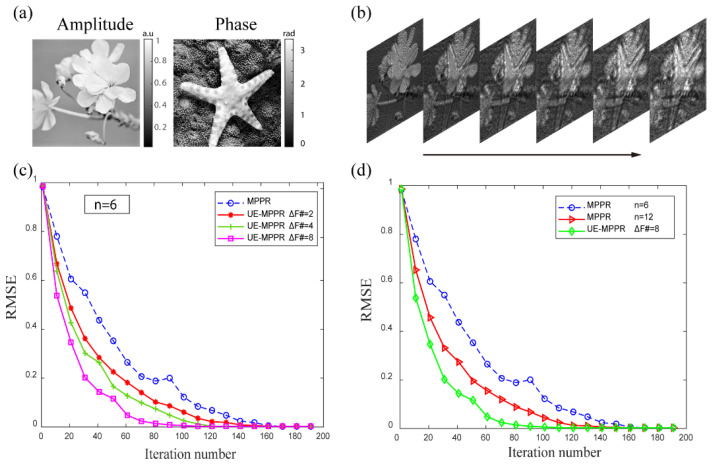
Simulations comparing phase retrieval methods. (**a**) Amplitude and phase of the simulated ground truth complex field. (**b**) The stack of propagation intensity images. (**c**) The RMSE plots of the MPPR and UE-MPPR methods with different interval spaces. (**d**) The RMSE plots of MPPR using 6 and 12 diffraction planes, and UE-MPPR using 5 diffraction planes with interval space of the adjacent planes $\Delta F^{\#}$ = 8.

We start with numerical simulations to verify the effectiveness of the proposed method, a series of simulations under different conditions were carried out and then the results were compared to the conventional MPPR algorithm. The ground truth complex field to be recovered is created from the images of “flower” and “starfish” for its amplitude and phase distribution, respectively, and its amplitude is normalized to values in the range 0–1 and the phase of values between 0 and π rad, the image has 200 × 200 pixels (pixel size 0.25 mm), as shown in Figure 2a. The incident wavelength is 1 mm, the initial distance from the first observation plane to the target object is $z_{0}=10$ mm, the number of the propagation plane(n) is 6 and the iteration number in the iterative algorithm is 200. The equal interval step between the adjacent planes in conventional MPPR is Δz = 30.3 mm, and in the proposed unequal interval method, the following 3 sets of interval steps are used: (1) $\Delta F^{\#}$ = 2, $\Delta z_{1}$ to $\Delta z_{5}$ are 25.00, 25.51, 26.04, 26.60, 27.17 mm, respectively; (2) $\Delta F^{\#}$ = 4, $\Delta z_{1}$ to $\Delta z_{5}$ are 25.00, 26.04, 27.17, 28.41, 29.76 mm, respectively; (3) $\Delta F^{\#}$ = 8, $\Delta z_{1}$ to $\Delta z_{5}$ are 25.00, 27.17, 29.76, 32.89, 36.76 mm, respectively.

First, the same number of recording planes is used for the two methods, and different $\Delta F^{\#}$ numbers for calculating the interval distance of the UE-MPPR method. For better comparison, the square root of MSE is calculated and normalized to [0 1], the RMSE curves of the retrieval images with different parameters are shown in Figure 2c. As the number of iterations increases, the RMSE values are gradually decreased. Obviously, the UE-MPPR algorithm with 3 sets of parameters converges faster. From Equation (4), we can see that when the $\Delta F^{\#}$ number increases, the distance between adjacent recording planes also increases. The 3 RMSE curves of the UE-MPPR algorithm show that the greater the distance between the adjacent surfaces, the better the recovery phase image quality. Subsequently, we selected the $\Delta F^{\#}$ = 8, which presents the best performance from the previous simulation experiment and used as the input parameters of the UE-MPPR method, we also increase the number of input recording planes of the conventional MPPR method. A previous study [19] has shown that the speed of convergence increases when more intensity planes are used. As can be seen from the MSE curve in Figure 2d, with the increase in the number of the input recording planes, the initial convergence speed of the conventional MPPR method is slightly faster than that of UE-MPPR. However, when the number of iterations exceeds 30, the advantage of convergence speed is negligible. As the number of iterations reaches 12, the convergence speed of the two algorithms is almost the same. To achieve the same image quality, the conventional MPPR algorithm uses nearly twice the number of input recording planes of UE-MPPR. Nevertheless, the greater number of diffraction planes means more images need to be measured, which may cause more measurement time consumption and noise accumulation in practical applications. Meanwhile, Figure 2c,d reveal that as the number of iterations increases, the quality of the image gets better, but it means more computations are required.

**Figure 3:**
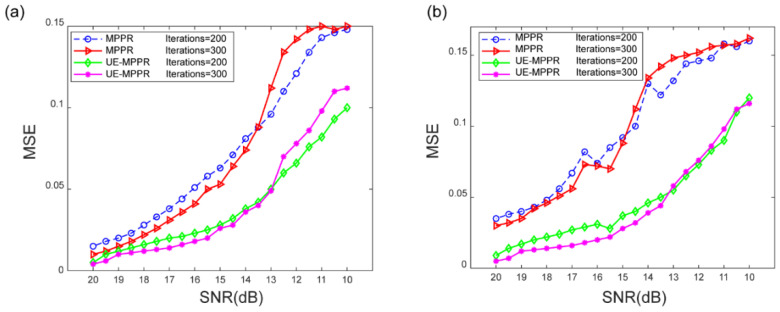
Comparison of mean square error (MSE) plots in phase results for various methods as the noise level increases. (**a**) The result by adding white noise. (**b**) The result by adding speckle noise.

To better evaluate the anti-noise performance of the proposed algorithm in practical applications, the intensity images of the equal and unequal spaced stacks are corrupted by two types of noise, one is the Gaussian white noise with SNR ranging from 20 to 10 dB, and the other one is the speckle noise. The MSE curve of the recovery phase image by adding noise is plotted in Figure 3, UE-MPPR shows more robustness than conventional MPPR in both noisy environments, especially when the SNR decreases to 14 dB, the conventional MPPR curve becomes fuzzy and the image cannot be restored. This fact indicated that the effect of more low-frequency information contained in the UE-MPPR recording process is good for the robustness of noise immunity. The plot also shows that when the noise increases to a certain value, with the number of iterations increasing, the image quality decreases slightly. This can be explained by the fact that the noise is always being accumulated during the iterative process of the algorithm. In practical applications, it is necessary to consider adding denoising processing and constraint methods [26,27,28] in the process of each iteration. Comparing the performance of the UE-MPPR and conventional MPPR in the two types of noisy environment, it can be found that the quality of the recovery image under Gaussian noise is better than that under speckle noise, the possible cause is that Gaussian noise is nearly uniformly distributed and is partially eliminated during the iteration process, while the distribution of speckle noise is more concentrated and cannot be eliminated in the iterative process, and may even be enhanced inversely, resulting in poor image quality.

**Figure 4:**
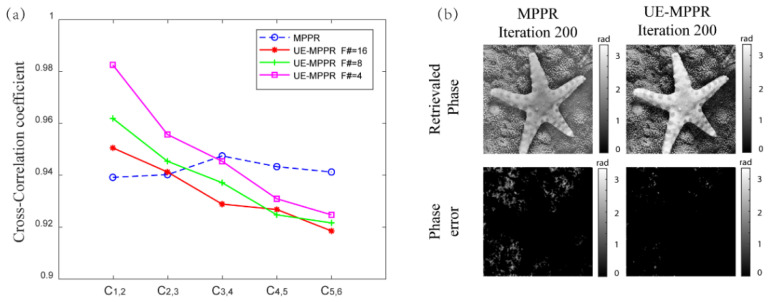
(**a**) The cross-correlation coefficient between the two adjacent planes using MPPR and UE-MPPR, respectively. (**b**) Recovered phase images and their errors compared to the true values by using the conventional MPPR and UE-MPPR methods.

To better explain the fast convergence and small reconstruction error achieved by UE-MPPR, the cross-correlation coefficient of the adjacent measured planes is calculated and shown in Figure 4a. $C_{i,i+1}$ represents the correlation coefficient between the plane $U_{i}$ and $U_{i+1}$. The smaller the correlation number is, the larger the strength difference between the adjacent planes, indicating that the diffraction pattern fully “regenerates” from one to another, which is a key and essential element for a successful iterative phase retrieval. Clearly, the coefficient curve is flat in the conventional MPPR algorithm (blue curve), which means that the variation in the measured intensity is very small, which may result in the stagnation of the iterative convergence. In contrast, in the enhanced UE-MPPR method (red, green, and magenta curve), the correlation coefficient margin between adjacent planes changes obviously from maximum to minimum and the large intensity difference of the adjacent planes originates from the unevenly spaced distances, which speeds up the convergence.

Besides the convergence speed, the quality of the recovery image is considered to evaluate the performance of the proposed algorithm. Figure 4b shows the residual graph of the image recovered by the conventional MPPR and UE-MPPR algorithms, respectively. The number of observation planes and the first record position z_0_ used in the two algorithms is the same, and the F number used in the UE-MPPR algorithm is $\Delta F^{\#}$ = 8. As can be seen from the image of “starfish”, the high frequency part of the recovery images by the two algorithms is basically the same, but there are some differences in the low frequency part. Images restored by the UE-MPPR algorithm can display more low-frequency information, which also explains the fact that UE-MPPR can not only collect more regeneration information but also the fact that the recovered phase image contributed low-frequency information. 

## 4. Experiment Results

**Figure 5:**
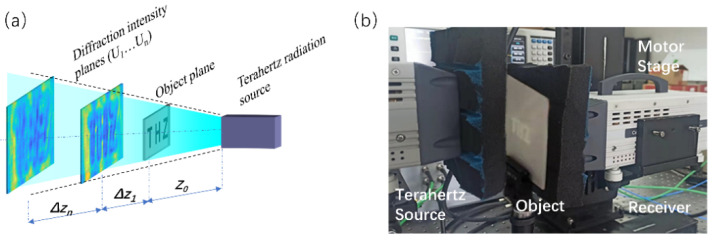
Terahertz lensless phase retrieval diagram (**a**) and experiment setup (**b**).

Experiments were carried out to further validate previously mentioned simulations. Figure 5 shows the experimental setup. A pair of vector network analyzer (VNA) frequency extenders (WR3.4SAX, 220–330 GHz from Virginia Diodes Inc., Charlottesville, VA, USA) combined with Keysight’s N9029A VNA forms a two-port network for transmitting and receiving terahertz waves, the working frequency of the terahertz wave is 300 GHz. The measured scattering parameter S21 (i.e., transmission coefficients) of the two-port network is utilized as the data input for the imaging reconstruction. A host computer is connected to the VNA via USB to collect data. Both the object and the transmitter are fixed and the object is perpendicular to the terahertz wave, the distance between the terahertz source and the object is *Z*_0_. The receiver is mounted on a three-dimensional translation motorized stage and records the image data in the raster scanning mode, the step of the *x* direction and *y* direction is the same, which obtains the recording of images at a size of 50 × 100 in 0.5 mm pixels. The receiver will shift programmatically to the next position along the *z* direction following the variation in Δz. The time for capturing an image is about half an hour. As mentioned in [29], the error that affects the recovery image can be ignored when the registered error is less than 0.2 pixels. The positioning accuracy of the 3D mobile platform is 2 μm, which promises the positioning accuracy of raster scanning and recorded images in the target locations. The equal interval step between the adjacent planes of conventional MPPR is Δz = 30.3 mm, the difference in the Fresnel number $\Delta F^{\#}$ = 8, the Δz of the UE-MPPR is varied with 25.00, 27.17, 29.76, 32.89, 36.76 mm, respectively.

**Figure 6:**
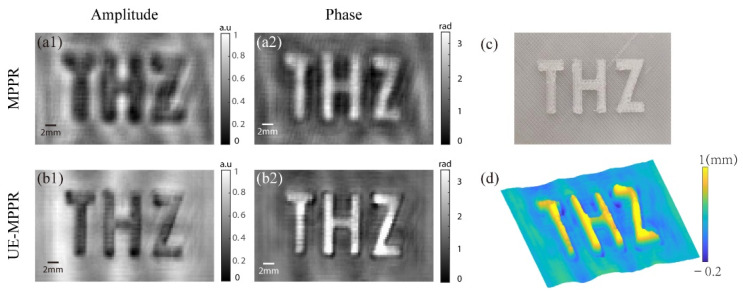
The reconstruction results of the 3D printing plate: (**a1**,**b1**) are the reconstructed amplitudes using MPPR and UE-MPPR method after 300 and 100 iterations, respectively; (**a2**,**b2**) are the corresponding reconstructed phases. (**c**) is the photo of the plate and (**d**) is the topography derived from (**b2**).

Figure 6a,b shows the recovery amplitude and phase images of the two methods with different iterations. A 3D-printed polylactic acid (abbreviated PLA, refractive index n_PLA_ = 1.41 at 0.3 THz) plate with a thickness of 2 mm, as depicted in Figure 6c, was used as a measured sample.In comparison, the UE-MPPR finished the phase recovery with 100 iterations while the conventional MPPR takes 300 iterations. Comparing the reconstruction quality, there are some vague parts in Figure 6a1 and some phase information is missing in Figure 6b1, the UE-MPPR achieves better clarity and contrast in both intensity and phase images. The average phase difference in the embossed pattern is 2.51 rad, corresponding to an average height of 1 ± 0.1 mm. Figure 6d is the topography extracted from Figure 6b2. Due to the low SNR of the terahertz system used in the experiment, recorded intensity images are corrupted during the data acquisition process, while UE-MPPR proves superior performance on noise resistance, most of the noise is suppressed in the iterative process. On the contrary, there are still many cloud-like artifacts in the image restored by the conventional MPPR, such artifacts are normally reduced by adding more record planes and denoise methods. Nevertheless, our experimental result reveals that UE-MPPR can use fewer recording planes to acquire a good recovery quality. In practical applications, fewer recording planes means less measurement time, which greatly expands the application field of this method.

In order to show the validity of the proposed method in imaging the phase object, the aforementioned experiment is repeated by using a step model made from high-density polyethylene (HDPE) as a tested object, the height of each step is 1 mm, and the other parameters are the same as the amplitude object measurement.

**Figure 7:**
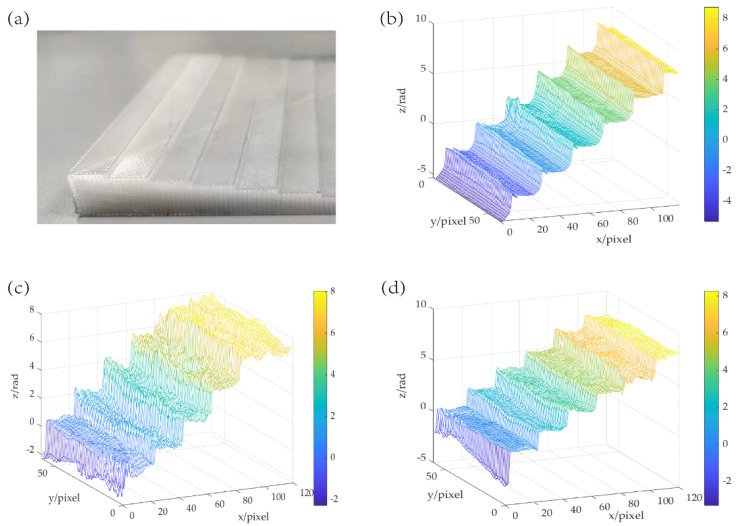
The phase distribution of the step captured. (**a**) Photo of the object (**b**) by RSFPI at 0.3 THz with NA = 0.24, (**c**) by conventional MPPR with 500 iterations and (**d**) by the UE-MPPR method with the $\Delta F^{\#}$ = 8, 300 iterations.

Figure 7 shows the recovery image of the two methods with different iterations. It can be seen that the conventional MPPR algorithm needs up to 500 iterations to reconstruct the phase image whilst the proposed UE-MPPR method only needs 300 iterations. As an empirical rule, the number of iterations used to restore the pure phase object is much higher than that of the amplitude object because the intensity change in the measurement is not significant enough, resulting in slow convergence. As can be seen from the quality of the phase image shown in Figure 7c, there are still some obvious defects that exist in the phase image recovered by the conventional MPPR algorithm, while the recovery image of the UE-MPPR method is smooth with a clear contour. Compared to the phase image obtained by the raster scanning focal plane imaging (RSFPI) (Figure 7b), there is more fluctuation between each adjacent step, especially on the edge of the step. We calculated the height of the steps from the phase image, each step height of the object calculated from the phase image is 1000+/−200 μm, which is in good agreement with the actual object.

## 5. Conclusions

In this paper, we have proposed a robust and accelerated terahertz multi-plane phase retrieval method to overcome the disadvantage of slow convergence or stagnation in the conventional multi-plane phase retrieval method. We created a nonlinearity spaced measurement scheme that significantly increases the diversity of the intensity with less images, in which the interval space between measurement planes is increasingly varied. We have demonstrated the proposed UE-MPPR method with both numerical simulation and measured experiments. Compared to the conventional MPPR method, our method can effectively improve the convergence speed and enhance the quality of reconstruction. Moreover, it is also robust and less sensitive to noise. The potential application is terahertz rapid phase imaging based on a simple and compact setup. Future work will focus on reducing the noise measurement and improving the calculation efficiency and quality of the reconstructed image.

## Data Availability

Not applicable.
